# Bimelic symmetric Hirayama disease: Spectrum of magnetic resonance imaging findings and comparative evaluation with classical monomelic amyotrophy and other motor neuron disease

**Published:** 2017-07-06

**Authors:** Deb Kumar Boruah, Shantiranjan Sanyal, Arjun Prakash, Sashidhar Achar, Dhabal D. Dhingani, Binod Sarma

**Affiliations:** 1 Department of Radio-Diagnosis, Assam Medical College and Hospital, Dibrugarh, Assam, India; 2 Department of Radiology, Airedale General Hospital, West Yorkshire, UK; 3 Department of Neurology, Assam Medical College and Hospital, Dibrugarh, Assam, India

**Keywords:** Monomelic Amyotrophy, Wasting, Lamino-Dural Space, Anterior Horn Cells, Amyotrophic Lateral Sclerosis

## Abstract

**Background:** The aim of the study was to evaluate the magnetic resonance imaging (MRI) findings in bilateral symmetrical Hirayama disease and find out MRI features which are probably more indicative of symmetrical Hirayama disease, thereby help in differentiating this entity from other motor neuron disease (MND).

**Methods:** This prospective as well as retrospective study was carried out from December 2010 to September 2016 in a tertiary care center of northeast India on 92 patients with Hirayama disease. Only 19 patients having bilateral symmetric upper limb involvement at the time of presentation were included in this study sample.

**Results:** Nineteen patients, who constituted 20.6% of 92 patients of clinical and flexion MRI confirmed Hirayama disease were found to have bilateral symmetrical wasting and weakness of distal upper limb muscles at the time of presentation. Mean ± standard deviation (SD) age of onset of the disease process was 21.7 ± 3.8 years with mean ± SD duration of illness of 3.6 ± 1.3 years. MRI revealed lower cervical cord flattening in 13 (68.4%) patients which was symmetrical in 6 (31.6%) patients and asymmetrical in 7 (36.8%) patients. In the majority of these patients, T2-weighted images (T2WI) cervical cord hyperintensities were found extending from C5 to C6 vertebral level. Seven (36.8%) patients in our study showed bilateral symmetric T2WI hyperintensities in anterior horn cells (AHC).

**Conclusion:** Bilateral symmetrical involvement of Hirayama disease is an uncommon presentation. Symmetrical cervical cord flattening, T2WI cord and/or bilateral AHC hyperintensities were the major MRI findings detected. Flexion MRI demonstrated similar findings in both bimelic amyotrophy and classical unilateral amyotrophy. However, flexion MRI produced some distinguishing features more typical for bilateral symmetrical Hirayama disease which help to differentiate it from other MNDs.

## Introduction

Hirayama disease was initially described by Hirayama, et al.^1^ in 1959 in a Japanese patient with atrophy of the distal upper limb also known as juvenile muscular atrophy of distal upper limb extremity^2^ or monomelic amyotrophy.^3^ Hirayama disease is characterized by insidious onset of asymmetric oblique amyotrophy characterized by wasting and weakness of distal muscles of upper extremity, predominantly affecting small muscles of hand in young (men > women) due to involvement of C7, C8 and T1 segmental myotomes with sparing of brachioradialis and proximal muscles of upper limb innervated by C5-C6 myotomes.^1^ Affection of lower limb muscles is very rare.^4^^,^^5^

Unilateral presentation is the most common in Hirayama disease, though few cases with asymmetrical involvement and rarely bilateral symmetrical involvement have been reported in literature.^6^^,^^7^ Various literatures have reported different pattern of disease onset and progression. The most rare type is bilateral symmetrical involvement which is seen in only 3.1% of Hirayama disease and which has an association with high level of serum immunoglobulin E.^8^^-^^10^ The bilateral symmetric Hirayama disease should be differentiated from diseases like other motor neuron diseases (MNDs), syringomyelia, spinal cord tumor, poliomyelitis, toxic neuropathies and traumatic myelopathy. These disorders can be differentiated from bilateral Hirayama disease with clinical, imaging features and genetic testing;^11^ whereas syringomyelia, spinal cord tumor or other space occupying lesions can be detected by spinal magnetic resonance imaging (MRI). 

We aimed to evaluate the MRI findings in bilateral symmetrical Hirayama disease and find out MRI features which help to differentiate this condition from other MNDs.

## Materials and Methods

Our data analysis was designed and the results were tabulated in keeping with a similar study by Pradhan, et al.^6^ including a large sample of symmetrical disease. We have compared our results with study conducted by Pradhan, et al.^6^ and other similar literatures in the past. 

After approval from the institutional ethics review committee, a hospital-based cross-sectional study was conducted. Based on the clinical and flexion MRI criteria, a total of 92 patients with confirmed Hirayama disease were evaluated from December 2010 to September 2016 in a tertiary care center of northeast India. Out of 92 confirmed cases with Hirayama disease, only 19 patients had bilateral symmetrical weakness and wasting of distal upper limbs muscles at the time of presentation and were included in this study. All these patients were assessed through clinical, electrophysiological and radiological evaluation. Informed consent was obtained from patients prior to MRI scan.

***Patient selection:*** We included patients in whom flexion cervical spine MRI was performed. MRI scan was performed using 1.5 Tesla Siemens Magnetom Avanto B15 (Siemens Medical Systems, Erlangen, Germany). Motor and sensory nerve conduction velocities (NCS) and compound muscle action potential (CMAP) amplitudes of median and ulnar nerves were measured in the affected upper limbs.

***MRI protocols in patient with Hirayama disease:*** Imaging of cervical spine was initially performed with patient in neutral supine position with routine sagittal T2- and T1-weighted spin-echo, sagittal and coronal short tau inversion recovery (STIR), and axial T2, T1-weighted fast spin-echo and axial 2D T2-weighted gradient-echo (GRE, Me-2D) sequences. Sagittal spin-echo T1WI were acquired with 450-500/9-15 (repetition time/echo time) while sagittal T2-weighted images (T2WI) were obtained with 4000-4600/110-120 (repetition time/echo time) with 3 mm slice thickness. Axial 2D T2-weighted GRE image was obtained with 650-750/24-32 (repetition time/echo time) with flip angle of 24° to 28°. Flexion MRI of the cervical spine was obtained in 30-40 degree neck flexion. Post-gadolinium fat suppressed sagittal and axial T1WI of cervical spine were obtained in neck flexion with slice thickness of 3 mm. 

***Image analysis:*** MRI scans were analyzed for cervical curvature, cord flattening, cord atrophy, T2WI cord or anterior horn cells (AHC) hyperintensities. The maximum forward shifting of posterior dural sac or lamino-dural space (LDS) was measured in midline on post-gadolinium fat suppressed sagittal T1WI on flexion MRI. Besides anterior-posterior (AP) and transverse (TR) diameter of cervical cord was also obtained in axial images both in neutral and flexion MRI at the site of maximum forward shifting of posterior dural sac. The spinal canal diameters were measured both in neutral and flexion sagittal MRI images. The cervical spinal canal diameter on flexion MRI was measured at the maximum site of posterior dural sac forward shifting. In order to standardize measurement method and to minimize measurement error, each parameter was measured by two radiologists working independently. 

Data were presented in terms of percentage, mean and standard deviation (SD). Calculations were done using SPSS software (version 16, SPSS Inc., Chicago, IL, USA).

## Results

Out of 92 patients with clinical and MRI confirmed Hirayama disease, 19 patients (20.6%) had bilateral symmetrical wasting and weakness of distal muscles of upper limbs at the time of presentation. The mean age ± SD at the time of presentation and the onset of disease process was 21.7 ± 3.8 and 18.2 ± 3.0 years, respectively. The mean ± SD duration of the disease process was 3.6 ± 1.3 years. The man to women ratio was 18:1. Initial onset of disease process was bilateral symmetrical in 8 (8.7%) patients, initial unilateral amyotrophy progressing to bilateral amyotrophy in 11 (11.9%) patients and unilateral amyotrophy in 73 (79.3%) patients in our study sample of 92 patients. The time taken to affect the opposite upper limb was 1.2 ± 1.2 years in those patients with initial onset of unilateral amyotrophy. 

Seven out of 8 patients with initial onset of symmetric disease were clinically suspected to be motor neuron disease and another one patient was toxic myelopathy. Initial unilateral amyotrophy progressed to bilateral amyotrophy in 11 patients clinically suspected to be Hirayama disease (Table 1).

**Figure 1:**
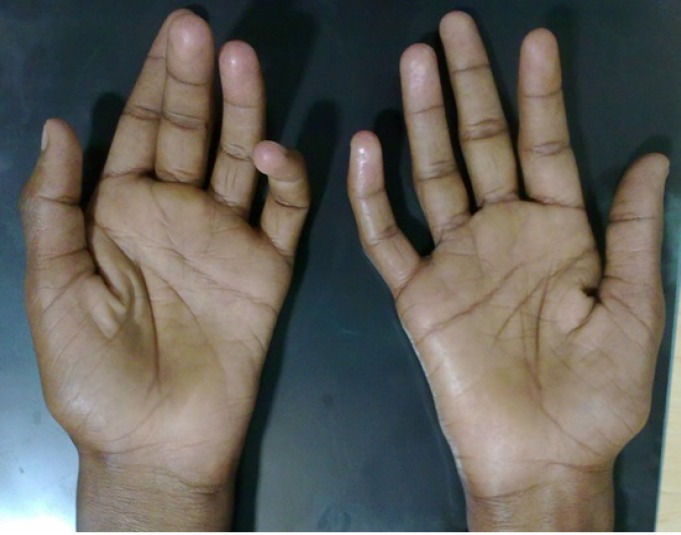
A 25-years old man with bilateral symmetrical wasting and weakness of hand muscles on examination showing flattened thenar and hypothenar eminences

All 19 (100%) patients had hand muscles wasting (Figure 1) and 6 (31.6%) patients also had wasting of forearm muscles. Sixteen (84.2%) patients had cold paresis in hands and 15 (78.9%) patients had hyperesthesia and fasciculation. Deep tendon reflexes of upper limbs were absent/hypoactive in 11 (58%), normal in 4 (21%) and brisk/hyperactive in 4 (21%) patients. The C7, C8 and T1 myotomes were involved in all 19 patients on NCS and electromyogram (EMG).

MRI revealed abnormal cervical curvature in 14 (73.7%) patients. Lower cervical cord flattening was noted in 13 (68.4%) patients which was symmetric in 6 (31.6%) patients and asymmetric in 7 (36.8%) patients. Seventeen (89.5%) patients had focal lower cervical cord atrophy, where cord atrophy extended from C5 to C6 vertebral level in 11 (57.9%) patients, C5 to C7 level in 5 (26.3%) patients and C6 to C7 vertebral level in 1 (5.3 %) patient (Table 2).

**Figure 2:**
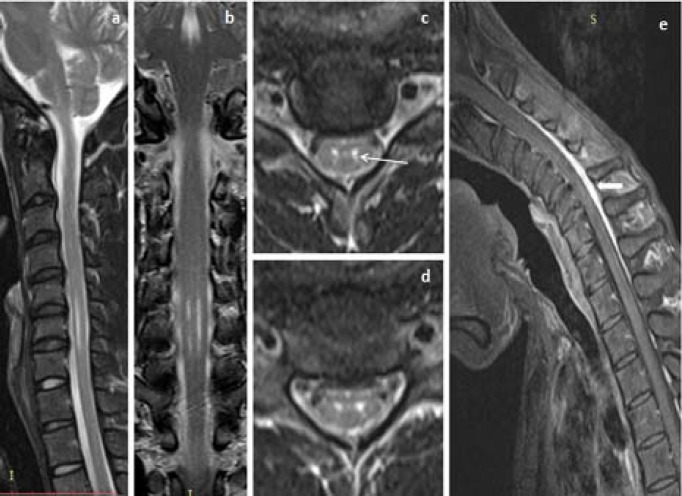
The same patient in figure 1, neutral position sagittal and coronal T2WI MRI images (a, b) showed segmental hyperintensities in lower cervical cord extending from C5 to C7 vertebral level, axial T2WI (c, d) showed symmetrical hyperintense signal in AHC (white arrow), post-gadolinium fat suppressed flexion MRI image (e) showed enhancing crescent shaped posterior epidural lesion extending from C4 to T2 vertebral level (block arrow)

**Figure 3:**
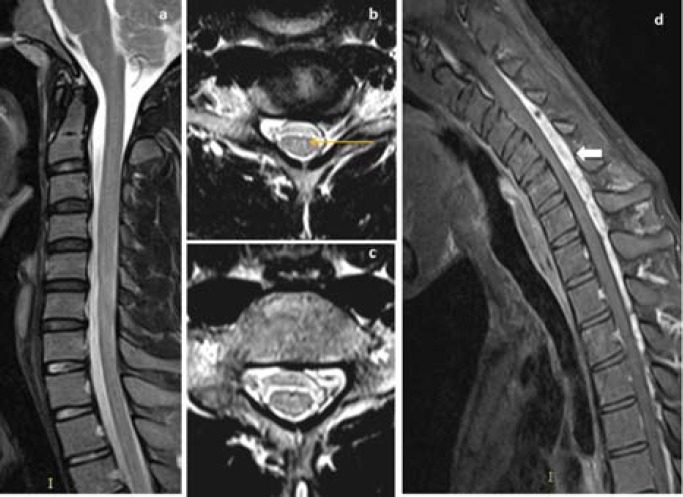
In a 19-year old man, neutral MRI sagittal T2WI (a) showed normal appearance of cervical cord without cord hyperintensities, axial T2WI (b, c) showed bilateral symmetrical faint T2W hyperintensities in AHC of lower cervical cord (yellow arrow), post gadolinium sagittal T1W flexion MRI image (d) showed enhancing posterior epidural lesion extending from C3 to T3 vertebral level (block arrow)

**Figure 4:**
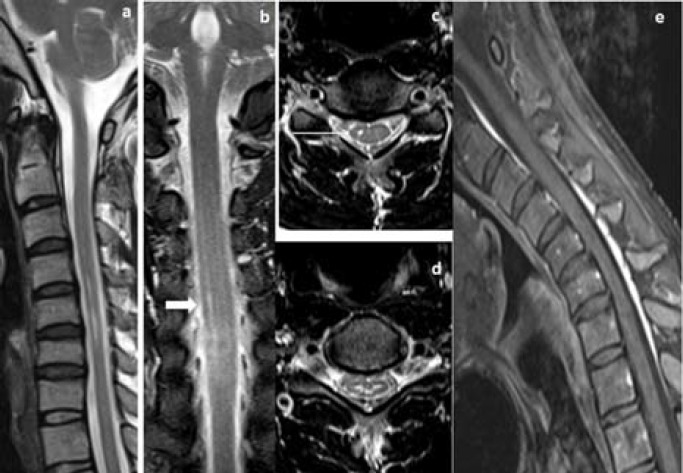
A 17-years old man presented with wasting of bilateral hand muscles. Neural position T2W sagittal and coronal MRI images (a, b) showed segmental hyperintensities in cervical cord extending from C4 to C6 vertebral level, more pronounced in right half of the cord (block arrow), axial T2WI (c, d) showed hyperintensities in bilateral AHC, more pronounced in right AHC and central cord substance (white arrow), post-gadolinium sagittal flexion MRI image (e) showed crescent shaped posterior epidural enhancing lesion with foreword shifting of posterior dural sac

**Figure 5:**
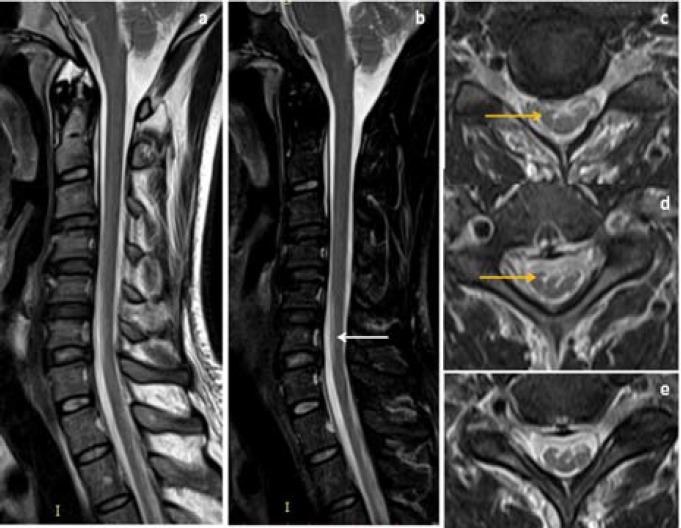
In a 18-years old man neutral MRI sagittal T2W and fat suppressed T2WI (a, b) showed lower cervical cord atrophy with hyperintensities in anterior cervical cord extending from C5 to C7 vertebral level, axial T2WI (c, d and e) showed asymmetrical hyperintensities in AHC (yellow arrows) and anterior cervical cord

Thirteen (68.4%) patients showed T2WI hyperintensities in lower cervical cord, which extended from C5-C6 vertebral level in 6 (31.68%) patients, C5-C7 in 5 (26.3%) patients, C6-C7 in 1 (5.3%) patient and C5-T1 vertebral level in another 1 (5.3%) patient (Table 2). T2WI hyperintensities in bilateral AHC giving ‘eye of snake’ appearance was demonstrated in 13 (68.4%) patients, where bilateral symmetrical AHC hyperintensities was seen in 7 (36.8%) patients (Figures 2 and 3), bilateral asymmetrical AHC hyperintensities, more pronounced in right AHC in 5 (26.3%) patients (Figures 4 and 5), and bilateral asymmetrical AHC hyperintensities, more pronounced in left AHC in 1 (5.3%).

**Figure 6:**
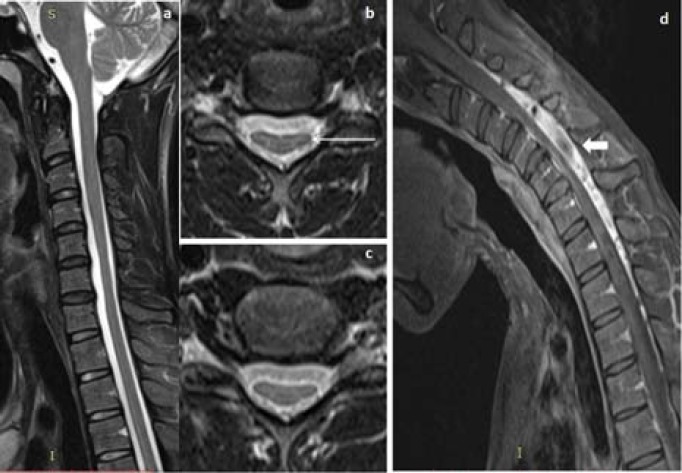
A 21-years old man with bilateral hand muscles weakness, neutral MRI sagittal T2WI (a) showed focal lower cervical cord atrophy without cord hyperintensities. Axial T2WI (b, c) showed anterior flattening of lower cervical cord (arrow), post-gadolinium sagittal flexion MRI T1WI (d) showed anterior displacement of posterior dura matter with enhancing posterior epidural lesion and T1 hypointense cerebrospinal fluid flow voids within (block arrow)

Flexion cervical MRI showed loss of dural attachment, forward shifting of posterior dural sac and post-gadolinium enhancing posterior epidural crescent shaped component due to engorged epidural venous plexus in all (100%) patients. The location of enhancing posterior epidural space component varied from C3 to T6 vertebral level, where 9 (47.4%) patients had in cervical region and 10 (52.6%) patients in cervico-dorsal region (Figures 3 and 6). 

**Table 1:** Summary of 19 patients with bilateral symmetric Hirayama disease at the time of presentation

**Case ** **no**	**Age at ** **presentation ** **(years)/gender**	**Age at ** **onset ** **(years)**	**Side of ** **initial ** **affection**	**Time to ** **affect ** **opposite ** **upper ** **limb ** **(years)**	**Focal ** **cervical ** **cord ** **atrophy**	**Cord ** **flattening**	**Level of ** **T2WI ** **cervico-dorsal ** **cord ** **hyperintensity**	**T2WI ** **hyperintensities ** **in AHC**	**Extension ** **of ** **enhancing ** **posterior ** **epidural ** **component ** **on flexion ** **MRI**	**Associated ** **disco-** **osteophytic ** **lesion**	**Prominent ** **epidural ** **flow voids**	**LDS ** **distance**	**Ratio of ** **LDS/spinal ** **canal ** **diameter ** **in flexion ** **MRI**	**Ratio of ** **AP/TR ** **cord ** **diameter ** **during ** **flexion ** **MRI**	**Ratio of** **AP/TR ** **cord ** **diameter** **during ** **neutral ** **MRI**
1	25/Man	19	Bilateral upper limbs	-	C5-C6	Symmetrical	C5-C7	Bilateral symmetrical	C5-D2	Yes	No	4.4	0.39	0.37	0.48
2	30/Man	23	Right upper limb	4	C5-C6	Asymmetrical	C5-C6	Bilateral asymmetrical, more on right side	C5-D1	Yes	No	4.8	0.36	0.43	0.49
3	19/Man	16	Left upper limb	2	C5-C7	Asymmetrical	No	No	C5-D5	No	Yes	5.8	0.42	0.22	0.32
4	18/Man	14	Bilateral upper limbs	-	C5-C7	Symmetrical	C5-C6	Bilateral symmetrical	C4-C6	No	No	3.5	0.27	0.32	0.41
5	19/Man	16	Right upper limb	2	C5-C7	No	No	No	C5-D5	No	Yes	9.8	0.71	0.31	0.45
6	16/Woman	14	Bilateral upper limbs	-	C5-C7	Symmetrical	C5-C7	Bilateral symmetrical	C4-C6	Yes	No	4.8	0.44	0.27	0.38
7	24/Man	19	Bilateral upper limbs	-	C5-C6	Symmetrical	C5-C7	Bilateral symmetrical	C5-C7	No	No	4.8	0.37	0.28	0.37
8	23/Man	20	Bilateral upper limbs	-	C5-C6	Symmetrical	C5-C6	Bilateral symmetrical	C5-C7	No	No	3.0	0.24	0.23	0.29
9	21/Man	18	Bilateral upper limbs	-	C6-C7	Symmetrical	C5-C7	Bilateral symmetrical	C4-T1	No	Yes	7.1	0.5	0.22	0.28
10	18/Man	16	Bilateral upper limbs	-	C5-C6	No	No	No	C5-C7	No	No	5.2	0.38	0.23	0.29
11	17/Man	14	Left upper limb	2	No	Asymmetrical	C5-C6	Bilateral asymmetrical, more on left side	C5-C7	No	Yes	4.7	0.35	0.28	0.31
12	19/Man	15	Right upper limb	3	C5-C6	No	C6-C7	Bilateral asymmetrical, more on right side	C4-C6	Yes	No	4.3	0.31	0.26	0.29
13	21/Man	19	Right upper limb	1	No	No	C5-T1	Bilateral asymmetrical, more on right side	C5-C7	No	No	6.1	0.42	0.34	0.38
14	21/Man	18	Right upper limb	1	C5-C6	Asymmetrical	No	No	C3-T2	No	Yes	8.4	0.69	0.21	0.32
15	20/Man	17	Right upper limb	2	C5-C6	Asymmetrical	No	No	C5-T1	No	No	5.2	0.46	0.35	0.46
16	26/Man	22	Right upper limb	2	C5-C7	Asymmetrical	C5-C7	Bilateral asymmetrical, more on right side	C5-T2	Yes	No	4.79	0.41	0.23	0.36
17	28/Man	24	Bilateral upper limbs	-	C5-C6	No	C5-C6	Bilateral symmetrical	C5-T6	No	No	3.9	0.29	0.27	0.35
18	25/Man	21	Right upper limb	2	C5-C6	No	C5-C6	Bilateral asymmetrical, more on right side	C4-C7	No	No	4.1	0.34	0.24	0.37
19	23/Man	20	Left upper limb	1	C5-C6	Asymmetrical	No	No	C5-T3	Yes	Yes	4.59	0.37	0.33	0.43

T2WI: T2-weighted image; AHC: Anterior horn cells; LDS: Lamino-dural space; AP: Anterior-posterior; TR: Transverse; MRI: Magnetic resonance imaging

**Table 2:** Magnetic resonance imaging (MRI) findings in 19 patients with bimelic symmetric Hirayama disease

**MRI finding**	**n (%)**
Abnormal cervical curvature	14/19 (73.7)
Cord flattening	13/19 (68.4) Symmetrical 6/19 (31.6) Asymmetrical 7/19 (36.8)
Location of cord atrophy	C5-C6 = 11/19 (57.9) C6-C7 = 1/19 (5.3) C5-C7 = 5/19 (26.3)
T2WI cord hyperintensity	C5-C6 = 6/19 (31.7) C6-C7 = 1/19 (5.3) C5-C7 = 5/19 (26.3) C5-D1 = 1/19 (5.3)
AHC hyperintensities	13/19 (68.4) Symmetrical 7/19 (36.8) Bilateral asymmetrical more on right 5/19 (26.3) Bilateral asymmetrical more on left 1/19 (5.3)
Level of enhancing epidural component extension	Cervical 9/19 (47.4) Cervico-dorsal 10/19 (52.6)
Flow voids in epidural component	6/19 (31.6)
Associated disco-osteophytic lesions	6/19 (31.6)

T2WI: T2-weighted image; AHC: Anterior horn cells; MRI: Magnetic resonance imaging

More common location of enhancing epidural component in C5-C7 vertebral level in 5 (26.3%) patients and C4-C6 vertebral level in 3 (15.8) patients. Prominent cerebrospinal fluid flow voids were noted within the enhancing posterior epidural component in 6 (31.6%) patients. Six (31.6%) patients with symmetrical Hirayama disease had also cervical disco-osteophytic bulges (Table 2).

The mean ± SD, LDS distance was 5.2 ± 1.7 mm at maximum forward shifting of posterior dura from the enhancing engorged epidural venous plexus in posterior epidural space. The ratio of maximum forward shifting of posterior dural sac (LDS)/maximum AP diameter of spinal canal during flexion MRI had an average increment value of 0.41 ± 0.12 mm which resulted in cord compression during flexion MRI. 

The ratio of AP/TR diameter of cord decreases during flexion MRI because of cord compression and cord flattening. 

The substantial decrement in AP/TR ratio diameter of cord during flexion MRI was 0.28 ± 0.06 (Table 3). Clinical and radiological data of all patients are summarized in table 1.

## Discussion

The initial symptoms of Hirayama disease are slowly progressing hand weakness and fatigue followed by cold paresis, tremors and atrophy. Asymmetric distribution of symptoms and signs is characteristic, although a bilaterally symmetric form has also been less frequently reported.^6^^,^^7^^,^^9^^,^^12^ Segmental C7-T1 myotomes involvement was seen in all cases of our study. The pathology for development of Hirayama disease is thought to be due to compression of lower cervical cord by the posterior dural sac during repeated or sustained neck flexion causing micro-circulatory changes in anterior spinal artery territory which further leads to degeneration of the AHC.^13^ Another pathogenetic mechanism cited for development of Hirayama disease is believed to be an imbalance growth between the individual vertebral column and spinal canal contents, leading to abutment of anterior spinal cord against vertebral column and detachment of the posterior dura leading to widened LDS and finally causing microcirculatory disturbances and ischemic changes in the anterior spinal cord.^14^^-^^17^

Lehman, et al.^18^ found that the median forward shift of posterior dura was 3 mm with mean of 2.7 mm (range 0 to 7 mm) in North American patients with Hirayama disease during flexion cervical MRI. In our study the median foreword shift of posterior dura was 4.8 mm with mean 5.2 mm (range 3 to 9.8 mm) during flexion cervical MRI.

**Table 3:** Measured parameters during neutral and flexion Magnetic resonance imaging (MRI) in 19 patients of bimelic symmetric Hirayama disease

**Parameters**	**Minimum**	**Maximum**	**Mean ± SD**
LDS distance (mm)	3.00	9.80	5.2253 ± 1.65862
Spinal canal diameter neutral (mm)	11.10	14.20	12.7158 ± 0.95176
Spinal canal diameter flexion (mm)	10.90	14.60	12.8474 ± 1.08031
AP cord diameter neutral (mm)	3.60	6.40	4.7889 ± 0.84989
TR cord diameter neutral (mm)	10.30	14.60	13.0053 ± 1.06847
AP cord diameter flexion (mm)	3.20	5.60	4.1658 ± 0.73637
TR cord diameter flexion (mm)	12.90	16.10	14.7895 ± 0.80270
LDS/spinal canal diameter flexion	0.24	0.71	0.4066 ± 0.12202
AP/TR cord diameter flexion	0.21	0.43	0.2837 ± 0.06039
AP/TR cord diameter neutral	0.28	0.49	0.3700 ± 0.06782

LDS: Lamino-dural space; AP: Anterior-posterior; TR: Transverse; SD: Standard deviation

Forward shifting of the posterior dural sac is also observed in normal subjects, but without spinal cord compression. The ratio of LDS at maximum forward shift to spinal canal diameter should be increased in Hirayama disease with decreased ratio of AP diameter of spinal cord to transverse diameter of spinal cord in flexion MRI compared to that in neutral position in Hirayama disease. These ratios do not significantly change in normal healthy subjects.^19^ In our study sample of 19 patients with bilateral Hirayama disease, the ratio of maximum forward shifting of posterior dural sac (LDS)/maximum AP diameter of spinal canal during flexion MRI had an average increment value of 0.41 ± 0.12 mm which ensured cord compression during flexion MRI. 

The ratio of AP/TR diameter of cord during neutral MRI was 0.39 ± 0.07 mm and decreased during flexion MRI because of cord compression and cord flattening with substantial reduction in ratio of AP/TR diameter of cord during flexion MRI (0.28 ± 0.06 mm, Table 3).

Zhou, et al.^20^ studied 192 patients with Hirayama disease in mainland China and found bimelic Hirayama disease in 25 patients (13%). In our study, bimelic Hirayama disease was noted in 19 patients out of 92 (20.6%) and initial onset of bilateral symmetric disease was noted in 8 (8.7%) patients followed by initial unilateral amyotrophy which progressed to bilateral amyotrophy in 11 (11.9%).

Pradhan^6^ evaluated 11 patients with bilateral symmetric Hirayama disease from North India and observed band like cord flattening on the MRI of all patients (100%) which was symmetric in 7 patients (63.6%) and asymmetric in 4 patients (36.4%). In our study sample of 19 patients, symmetrical and asymmetrical lower cervical cord flattening was noted in 6 (31.6%) and 7 (36.8%) patients, respectively.

Preethish-Kumar, et al.^12^ observed bilateral AHC T2WI hyperintensities giving “snake eye” appearance in 65.4% cases in South India, where 57.7% cases showed bilateral symmetrical AHC hyperintensities from. In our study, bilateral AHC T2WI hyperintensities were noted in 68.4% cases, symmetrical in 36.8% cases, asymmetrical with more pronounced signal in right AHC in 26.3% of cases and bilateral asymmetrical hyperintensities more pronounced on left AHC in 5.3% of cases.

Pradhan^6^ observed inferior extension of crescent shaped enhancing epidural component during post gadolinium flexion MRI up to the level of T2 vertebral body, however in our study, 10 (52.6%) patients had inferior dorsal extension of enhancing posterior epidural component during flexion MRI, where 2 (10.5%) patients had inferior extension up to T5 vertebral level and 1 patient (5.3%) up to T6 vertebral level. 

The typical MRI findings in Hirayama disease may reveal atrophy of lower cervical cord, asymmetric cord flattening, and/or T2WI cord hyperintensities. On neck flexion MRI, anterior displacement of the dorsal dura may be seen with crescent post contrast enhancing venous plexus engorgement in posterior epidural space.^13^

In other MND with spinal cord involvement, progressive neuronal degenerations occur along the corticospinal tract (CST) in the spinal cord. In the most common MND like amyotrophic lateral sclerosis (ALS), the initial MRI findings reveal symmetrical T2WI hyperintensities in anterior lateral column of spinal cord along the CST with preservation of posterior lateral column.^21^ It may also show bilateral symmetrical T2WI hyperintensities along AHC giving “snake eye” appearance and in this situation it is difficult to differentiate on conventional MRI from the bilateral symmetric Hirayama disease. However, flexion cervical MRI helps to differentiate as ALS does not reveal anterior displacement of dorsal dura or enhancing posterior epidural lesion. In patients with ALS, brain MRI shows bilateral T2WI hyperintensities extending along CST from centrum semiovale through posterior limb of internal capsule to ventral brain stem.^21^^,^^22^

In patients with spinal muscular atrophy (SMA), T2WI cord hyperintensities may be present in AHC of spinal cord associated with denervation atrophy of axial or proximal muscles. Proximal muscles are dominantly affected in SMA compared to distal muscles. However excessive fatty infiltrations of muscle bundles and increased intermuscular fat planes is noted in SMA. Lower limbs are more affected than upper limbs.^23^

As Hirayama disease has a self-limiting course, the treatment is usually conservative. The treatment involves reducing repeated trauma to cervical cord by avoiding repeated neck flexion, use of soft cervical collar during progressive stage of the disease which has shown to arrest the disease progression.^24^ Even surgical interventions like cervical decompression and fusion with or without duraplasty or cervical duraplasty with tenting sutures via laminoplasty without cervical fusion may be advocated in selected patients.^25^^,^^26^

Hence early recognition of Hirayama disease is necessary since avoiding or limiting neck flexion prevents or arrest further progression of this disease. So, a high clinical suspicion is necessary to diagnose the bimelic Hirayama disease, and include flexion MRI in addition to neutral MRI while imaging such patients. MRI may help in differentiating bilateral Hirayama disease from other MND, syringomyelia and neuropathies. 

Both monomelic and bimelic amyotrophy show similar findings during flexion MRI; however, conventional MRI findings of symmetrical lower cervical cord flattening and bilateral symmetrical AHC hyperintensities favor bimelic amyotrophy. In situations like absence of T2WI cord or AHC hyperintensities, radiological differentiation between monomelic and bimelic Hirayama disease is difficult.

## Conclusion

Bilateral symmetric involvement in Hirayama disease, an uncommon occurrence, is usually underdiagnosed because of a common understanding that Hirayama disease has unilateral or asymmetric bilateral involvement. Early diagnosis of bilateral symmetrical disease might help in limiting further progression of the disease by simple means such using cervical collar and asking patient to restrict neck flexion movements.

Conventional MRI findings like symmetrical cord flattening/atrophy and symmetrical T2WI hyperintensities in cord and or AHC favor bilateral symmetric Hirayama disease; however it is essentially difficult to differentiate bilateral symmetrical amyotrophy from the more common classical unilateral amyotrophy through imaging only.

## Notes:

**How to cite this article:** Boruah DK, Sanyal S, Prakash A, Achar S, Dhingani DD, Sarma B. Bimelic symmetric Hirayama disease: Spectrum of magnetic resonance imaging findings and comparative evaluation with classical monomelic amyotrophy and other motor neuron disease. Iran J Neurol 2017; 16(3): 136-45.
